# Cardiometabolic and immune response to exercise training in patients with metabolic syndrome: retrospective analysis of two randomized clinical trials

**DOI:** 10.3389/fcvm.2024.1329633

**Published:** 2024-04-03

**Authors:** Katharina Lechner, Sylvia Kia, Pia von Korn, Sophia M. Dinges, Stephan Mueller, Arnt-Erik Tjønna, Ulrik Wisløff, Emeline M. Van Craenenbroeck, Burkert Pieske, Volker Adams, Axel Pressler, Ulf Landmesser, Martin Halle, Nicolle Kränkel

**Affiliations:** ^1^ Department of Prevention and Sports Medicine, University Hospital Klinikum Rechts der Isar, School of Medicine, Technical University of Munich, Munich, Germany; ^2^ DZHK, German Centre for Cardiovascular Research, Partner Site Munich Heart Alliance, Munich, Germany; ^3^ Klinik für Herz- und Kreislauferkrankungen, Deutsches Herzzentrum München, Technische Universität München, Munich, Germany; ^4^ Deutsches Herzzentrum der Charité, Klinik für Kardiologie, Angiologie und Intensivmedizin, Berlin, Germany; ^5^ DZHK, German Centre for Cardiovascular Research, Partner Site, Berlin, Germany; ^6^ Cardiac Exercise Research Group (CERG), Department of Circulation and Medical Imaging, Norwegian University of Science and Technology, Trondheim, Norway; ^7^ Centre for Research on Exercise, Physical Activity and Health, School of Human Movement and Nutrition Sciences, University of Queensland, Brisbane, Queensland, Australia; ^8^ Research Group Cardiovascular Diseases, University of Antwerp, Antwerp, Belgium; ^9^ Department of Cardiology, Antwerp University Hospital (UZA), Edegem, Belgium; ^10^ Department of Internal Medicine and Cardiology, Campus Virchow Klinikum, Charité Universitätsmedizin Berlin, Berlin, Germany; ^11^ Department of Cardiology and Internal Medicine, Heart Center Dresden-University Hospital, TU Dresden, Dresden, Germany; ^12^ Private Center for Sports and Exercise Cardiology, Munich, Germany; ^13^ Friede Springer—Centre of Cardiovascular Prevention at Charité, Charité University Medicine Berlin, Berlin, Germany

**Keywords:** metabolic sydrome, exercise training, cardiovascular risk factor control, inflammation, cardiorespiratory fitness

## Abstract

**Background:**

Metabolic syndrome (MetS) is defined by the presence of central obesity plus ≥two metabolic/cardiovascular risk factors (RF), with inflammation being a major disease-driving mechanism. Structured endurance exercise training (ET) may positively affect these traits, as well as cardiorespiratory fitness (V̇O_2_peak).

**Aims:**

We explore individual ET-mediated improvements of MetS-associated RF in relation to improvements in V̇O_2_peak and inflammatory profile.

**Methods:**

MetS patients from two randomized controlled trials, ExMET (*n* = 24) and OptimEx (*n* = 34), had performed 4- or 3-months supervised ET programs according to the respective trial protocol. V̇O_2_peak, MetS-defining RFs (both RCTs), broad blood leukocyte profile, cytokines and plasma proteins (ExMET only) were assessed at baseline and follow-up. Intra-individual changes in RFs were analysed for both trials separately using non-parametric approaches. Associations between changes in each RF over the exercise period (*n*-fold of baseline values) were correlated using a non-parametrical approach (Spearman). RF clustering was explored by uniform manifold approximation and projection (UMAP) and changes in RF depending on other RF or exercise parameters were explored by recursive partitioning.

**Results:**

Four months of ET reduced circulating leukocyte counts (63.5% of baseline, *P* = 8.0e-6), especially effector subtypes. ET response of MetS-associated RFs differed depending on patients’ individual RF constellation, but was not associated with individual change in V̇O_2_peak. Blood pressure lowering depended on cumulative exercise duration (ExMET: ≥102 min per week; OptimEx-MetS: ≥38 min per session) and baseline triglyceride levels (ExMET: <150 mg/dl; OptimEx-MetS: <174.8 mg/dl). Neuropilin-1 plasma levels were inversely associated with fasting plasma triglycerides (*R*: −0.4, *P* = 0.004) and changes of both parameters during the ET phase were inversely correlated (*R*: −0.7, *P* = 0.0001).

**Conclusions:**

ET significantly lowered effector leukocyte blood counts. The improvement of MetS-associated cardiovascular RFs depended on individual basal RF profile and exercise duration but was not associated with exercise-mediated increase in V̇O_2_peak. Neuropilin-1 may be linked to exercise-mediated triglyceride lowering.

## Introduction

Risk factors defining the metabolic syndrome (MetS) predispose to atherosclerotic cardiovascular disease (ASCVD) and type 2 diabetes mellitus (T2DM), which pose a significant burden to our societies and health systems (1, 2). According to the International Diabetes Federation (IDF), MetS is defined by the presence of central obesity and two or more of the following criteria: dyslipidaemia (elevated triglycerides, low HDL-C), elevated fasting plasma glucose (FPG) and/or elevated diastolic or systolic blood pressure (DBP, SBP, respectively), or their specific pharmacologic treatment (1). While the exact terminology and criteria of MetS have elicited debate, the underlying concept remains that certain cardio-metabolic risk factors often occur in combination and that this clustering of risk factors disproportionally increases cardio-metabolic risk.

Inflammation, which is bidirectionally linked to ectopic/central adiposity (3), is a central mediator of risk factor clustering in MetS: once the lipid storage capacity of visceral adipose tissue is exceeded, cellular stress responses lead to increased release of inflammatory cytokines, reactive oxygen species and other molecular patterns of cellular damage, which in turn activate innate immunity (4). While systemic inflammatory state and overall leukocyte activation are tightly interwoven with dysregulation of glucose handling and development of ASCVD risk factors, individual subsets of leukocytes may differentially reduce or increase cardiovascular risk (5–11). Considering the heterogeneity of individual risk factor profiles among people with MetS, it remains unclear whether certain imbalances in the spectrum of leukocyte subsets (e.g., cytotoxic vs. regulatory T lymphocytes, classical vs. non-classical monocytes) are a common denominator for MetS or are associated with specific risk factor constellations.

Individual cardiovascular and metabolic risk factors (dysglycaemia, dyslipidaemia and hypertension) should be treated according to the respective guidelines (12–15). New pharmacologic developments create hope for effective weight loss in these patients (16). Nevertheless, exercise training (ET) is required to maintain or increase lean (muscle) mass and additional mechanisms mediated by ET, including paracrine and endocrine metabolic effects of hormone-like substances (i.e., “myokines” secreted by the contracting skeletal muscle), improved vascular function, contribute to a reduction in cardiovascular and metabolic risk factors, including hypertension (17–19). Endurance ET is thus fundamental for risk factor management in patients with MetS and T2DM, although long-term adherence is crucial to its effect, as was observed in the Look AHEAD trial and others (12–15, 20–22). Most data are available for endurance training performed at continuous moderate intensity (MICT), but evidence is accumulating that also endurance exercise at high intensities—most often performed in intervals (HIIT)—can be used.

Heterogeneity in the risk factor profile is inherent to the definition of the metabolic syndrome (1). Therefore, we hypothesize that benefit from the exercise intervention differs depending on patients' individual underlying risk factor profile with respect to both, the severity and the composition of risk factors. Moreover, we aimed to assess whether different exercise parameters, such as intensity or duration, differ regarding their relevance for the improvement of individual risk factors in individuals with different underlying risk profiles. Secondary questions were changes in the inflammatory spectrum and plasma proteins with cardio-metabolic relevance, potentially associated with improvement in individual risk factors.

## Material and methods

**Figure 1:**
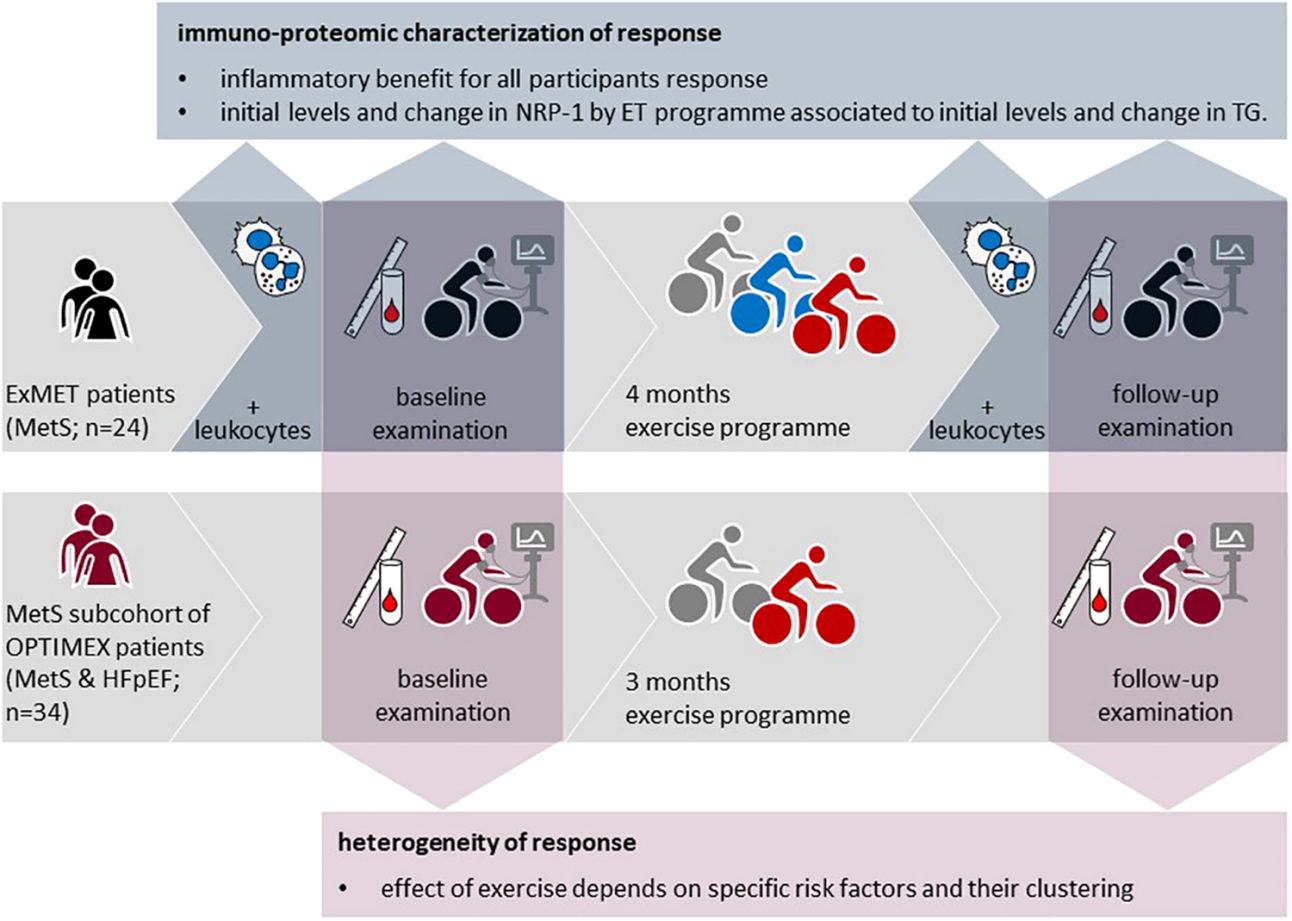
Study concept and origin of data from the OptimEx-clin and ExMET trials.

This is an exploratory, secondary analysis of the ExMET study and of the subgroup of patients with MetS from the OptimEx trial (Table 1, Figure 1).

**Table 1:** Patient characteristics at baseline.

	ExMET (*n* = 24)	OptimEx-MetS (*n* = 34)
Sex [% male]*	66.7	38.2
Age [years]*	62.0 (57.0–64.3)	70.5 (65.3–75.0)
Waist circumference [cm]	109.0 (96.6–112.3)	107.5 (100.3–118.5)
SBP [mmHg]	130.0 (120.0–140.0)	135.0 (121.3–140.0)
DBP [mmHg]*	85.0 (80.0–90.0)	76.5 (70.0–80.0)
HDL-C [mg/dl]	49.0 (40.0–57.0)	46.8 (41.9–53.9)
Fasting plasma triglycerides [mg/dl]*	125.0 (100.0–173.3)	177.6 (114.9–219.0)
Fasting plasma glucose [mg/dl]	106.5 (100.8–117.3)	111.0 (101.1–126.7)
V̇O_2_peak [ml/(min*kg)]*	22.1 (19.1–27.4)	16.6 (13.1–18.4)

Values are median (IQR) or %.

Chi square test was performed to compare sex distribution between both studies. Wilcoxon test was used to compare numerical parameters.

* *p* < 0.05 ExMET vs. OptimEx-MetS.

An expanded methods section is provided with the online supplement.

### Patient inclusion, clinical measurements, and exercise program

The studies complied with the declaration of Helsinki in its revised form of 2013 (23) and with Good Clinical Practice. The responsible ethics committees approved the study protocol of ExMET [Regional Committee for Medical Research Ethics (REK 2011/2150)] and OptimEx (403/13 ethics committee of the Technical University of Munich as well as each study site). All participants gave written informed consent prior to undergoing any study-related procedures. Both trials have been registered at ClinicalTrials.gov (NCT01676870, NCT02078947) and protocols have been published in advance (24, 25). Baseline clinical parameters of both trials are given in Table 1.

### ExMET intervention trial

Forty-eight patients (age 55–70 years, male and female) fulfilling IDF criteria for MetS were randomized (1:1:1) to a moderate intensity continuous training (MICT), low-, or high-volume high intensity interval training (HIIT) of 4 months duration. MICT was defined as two supervised sessions of 30 min continuous aerobic training at 35%–50% heart rate reserve (HRR) and three home-based exercise sessions, according to current guideline-based standard of care. Low-, and high-volume HIIT were defined as a 10-min warm-up phase at 35%–50% HRR, followed by a 4-min interval at 80%–90% HRR for the low-volume HIIT-group and four 4-min intervals for the high-volume HIIT-group in a supervised setting (Supplementary Figures S1A,B). The detailed protocol has been published and the study has been completed (26). Patient recruitment, assessment of clinical patient characteristics, exercise testing, blood sampling and risk factors and training were performed at the University Hospital Klinikum rechts der Isar, TU Munich during 2017 and 2018. Baseline and follow-up visits included a medical examination, anthropometric measurements, laboratory testing and standardized cardiopulmonary exercise testing (CPET). Venous blood samples were drawn from an antecubital vein after 12 h of fasting and after 24 h of abstinence from alcohol and vigorous exercise. The study investigators did not change medication (as prescribed by the treating physician) at baseline and patients were encouraged to take medications as prescribed throughout the study period. Twenty-four patients (MICT: *n* = 10, low volume HIIT: *n* = 9, high volume HIIT: *n* = 5) completed the training program with an adherence of at least 70% of sessions and we were able to obtain blood samples of sufficient quality for further analysis (Supplementary Figure S1C).

### MetS subgroup of the OptimEx intervention trial

Of the *n* = 180 male or female sedentary patients with heart failure with preserved ejection fraction (HFpEF as defined in (27); [exertional dyspnea (New York Heart Association class II–III), LVEF of 50% or greater, and elevated estimated LV filling pressure (E/e’ medial ≥15) or E/e’ medial of 8 or greater with concurrent elevated natriuretic peptides (NT-proBNP ≥220 pg/ml or BNP ≥80 pg/ml)] included into the completed OptimEx trial (28) during 2014–2018, *n* = 34 patients from the MICT (*n* = 17) and HIIT (*n* = 17) training arms meeting the IDF criteria for MetS (1) and with data of baseline and 3-month visit complete were included into this analysis (Supplementary Figure S1F). Patients from all sites of the OptimEx trial (Berlin, Leipzig, and Munich, Germany; and Antwerp, Belgium) were included into this analysis. MICT was defined as five sessions of 40 min continuous aerobic training at 35%–50% HRR. HIIT sessions lasted 38 min, composed of a 10-min warm-up phase at 35%–50% HRR, followed by four 4-min intervals at 80%–90% HRR interspaced by 4 min of active recovery (Supplementary Figures S1D,E). Patients were included in the OptimEx trial if heart failure with preserved ejection fraction (HFpEF), based on elevated natriuretic peptides, left ventricular ejection fraction of ≥50% and increased estimated left ventricular filling pressure (27) were evident and signed informed consent obtained. Examination at baseline and 3 months-visit included, amongst others, medical history, anthropometric measurements, blood sampling and CPET (28).

### Leukocyte profiling

One hundred microlitres of freshly withdrawn, EDTA-anticoagulated blood were stained with directly fluorochrome-labelled antibodies. Samples were measured within three days and diluted 5-fold in PBS prior to acquisition on an Attune NxT acoustic focusing cytometer (ThermoFisherScientific). Investigators were blinded to the group allocation of participants during measurement and gating.

### Microvesicle measurements

Platelet-depleted plasma was prepared by sequential centrifugation from freshly withdrawn ACD-anticoagulated blood and stored at −80°C. For analysis, plasma samples were thawed, centrifuged again at 2000×g for 20 min and stained with directly fluorochrome-labelled antibodies and Annexin V in Annexin V Binding Buffer (BioLegend) containing 20 mM CaCl_2_. Samples were acquired immediately on an Attune NxT acoustic focusing cytometer (Thermo Fisher Scientific). Investigators were blinded to the group allocation of participants during measurement and gating.

### Plasma measurements

Concentrations of cytokines (interleukin (IL)-1β, IL-8, IL-10, IL-13, basic fibroblast growth factor (FGF), interferon (IFN)-γ, IFN-γ induced protein (IP)-10, monocyte chemoattractant protein (MCP)-1, tumor necrosis factor (TNF)-α) in plasma samples of the ExMET cohort were assessed by the cytokine bead array (CBA) flex kits (BD) according to the protocol recommended by the manufacturer and read in Attune NxT acoustic focusing cytometer (Thermo Fisher Scientific).

A panel of 86 plasma proteins relevant to cardiometabolic disease was assessed in plasma samples of the ExMET cohort, but not the OptimEx-MetS cohort using proximity extension assay by Olink^©^ Proteomics (Uppsala, Sweden).

While the proteome panel was chosen by the authors due to its general relevance in innate immunity and cardio-metabolic dysfunction, individual analytes where not selected based on a pre-defined hypothesis. Cytokine and plasma protein analyses were not available for the MetS patients of the OptimEx population.

Apolipoprotein C3 plasma levels were assessed in plasma samples from the OptimEx-MetS-cohort using the Apolipoprotein C3 Human ELISA Kit (Invitrogen). Investigators were blinded to the group allocation of participants during measurement and calculation of concentrations.

### Statistics

Parameters or participants with more than 10% missings of the respective parameters were omitted from the analysis. Excel 2016 (Microsoft) was used to compile data and R version 3.6.0 with R Studio version 1.1.463 was used for analysis and drawing of graphs. Non-parametric tests were used at all times due to the low n. The Benjamini-Hochberg method was used to adjust for multiple testing and false discovery rate (FDR) is given. A *p*-value of less than 0.05 was considered statistically significant. “Delta” values (Δ) denote *n*-fold changes at the 3-month or 4-month follow-up time point, normalized to the baseline value of the same individual. Structure and relationships within data sets were explored and visualized by Uniform Manifold Approximation and Projection (UMAP) and Gaussian Mixture Model (GMM). Correlations between multiple parameters were visualized as a network with correlations indicated as lines between correlated parameters and parameters themselves were visualized as dots.

## Results

### Changes in V̇O_2_peak and MetS-defining cardiovascular risk factors by exercise programmes in two independent populations with MetS

**Figure 2:**
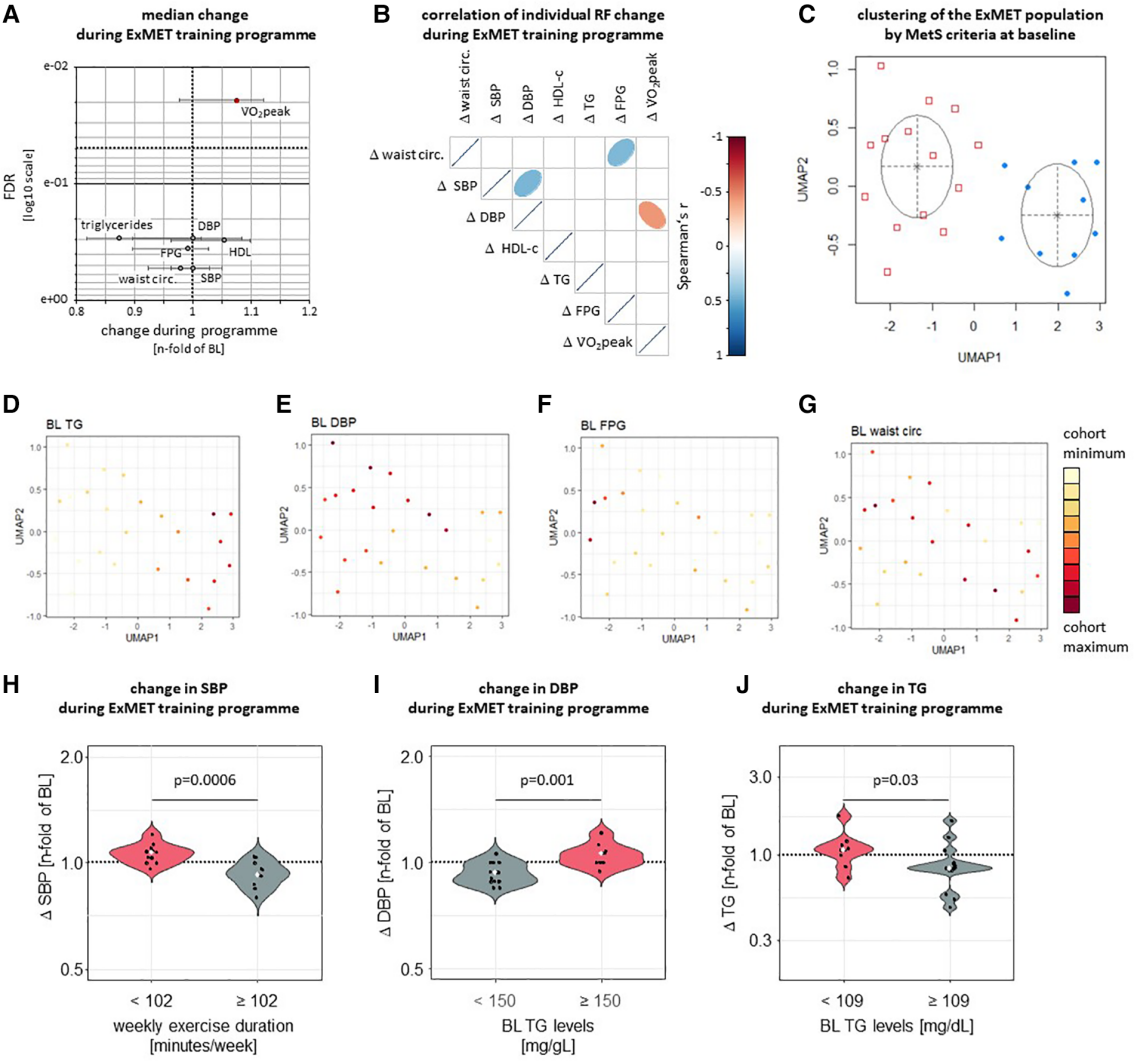
Only V̇O_2_peak was significantly improved by a 4-month exercise intervention in the ExMET study (**A**, data are median with IQR). Changes in V̇O_2_peak (adjusted for body weight) during the ET program correlated weakly with changes in DBP and did not correlate with changes in FPG, plasma lipids or central obesity (**B**) UMAP was used to reduce dimensions based on waist circumference, HDL-C, TG, FPG, SBP and DBP at baseline with GMM used for identification of clusters (**C**). Distribution of individual MetS parameters within clusters indicates differential risk profiles defined by fasting triglyceride levels (**D**; *p* = 4.64e-05 between clusters) and DBP (**E**; *p* = 0.018 between clusters), but not fasting glucose or waist circumference (**F**, **G**, *p* > 0.05 between clusters). Colour range indicates distribution within whole study population. SBP was reduced in participants reaching a cumulative exercise duration of at least 102 min per week (**H**) DBP was lowered more effectively in participants with lower TG levels at baseline (**I**) and relative TG lowering was greater in participants with higher TG levels at baseline (**J**).

Within the ExMET population (*n* = 24), V̇O_2_peak increased during the course of the 4-month ET program (24, 26), but other MetS-defining parameters—waist circumference, fasting triglycerides, HDL-C, SBP and DBP—did not change significantly (Figure 2A). Improvements in V̇O_2_peak during the ET program correlated weakly with reductions in DBP, but not with any other MetS parameter (Figure 2B) and were not associated with the ET modality (26).

Heterogeneity in the risk factor profile is inherent to the definition of the metabolic syndrome (1) and we hypothesized that benefit from the exercise intervention differs depending on patients’ underlying individual risk factor profile. We therefore used Uniform Manifold Approximation and Projection (UMAP) (29) to explore individual risk factor clustering within the ExMET cohort. Two clusters of patients, mainly differing in TG levels (*p* = 4.6e-05) and DBP (*p* = 0.02), were identified by Gaussian Mixture Model (GMM) analysis (Figures 2C–G, Supplementary Figure S2). In addition, we used recursive partitioning to explore the potential role of MetS risk factor at inclusion and parameters of the training modality for the improvement of each individual MetS parameter during the exercise program (Supplementary Figure S3). SBP reduction was greater in patients exercising for more than 102 min per week, than in patients exercising for a shorter duration (Figure 2H), while improvement in DBP and TG differed in patients with higher vs. lower TG levels at baseline (Figures 2I,J), using cut-offs as calculated by recursive partitioning (Supplementary Figure S3). When comparing pre-specified training protocols (MICT, low volume HIIT, high volume HIIT) we had previously not observed differences in the change of cardiometabolic parameters (26).

**Figure 3:**
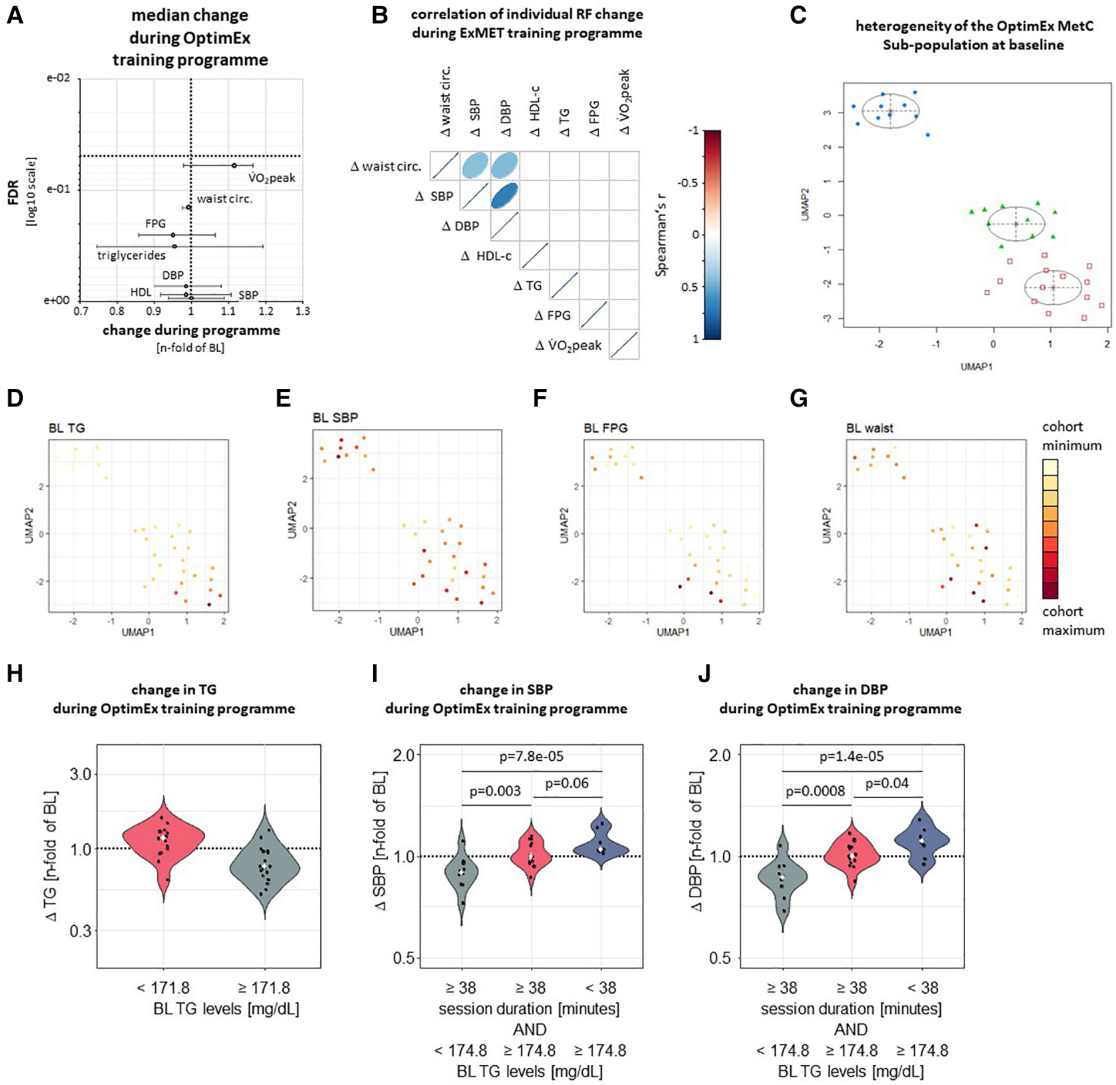
Following adjustment for multiple testing, neither V̇O_2_peak nor MetS-defining risk factors significantly improved during three-months supervised exercise training within the OptimEx-MetS cohort (**A**). Change in V̇O_2_peak during a three-month supervised exercise program did not correlate with changes in risk factors defining the Metabolic Syndrome (MetS) in the OptimEx cohort (**B**). UMAP followed by cluster identification by GMM identified three clusters of patients differing in their risk factor profile at baseline (**C**). Clusters differed from each other mainly in baseline TG levels (**D**, *p* = 4.9e-07), SBP (**E**, *p* = 0.04) and FPG (**F**, *p* = 0.02), but not in waist circumference (**G**, *p* > 0.05 between clusters). Colour range indicates distribution within whole study population. Relative TG lowering was greater in participants with higher TG levels at baseline (**H**). SBP and DBP reduction was more effective in participants reaching a cumulative exercise duration of at least 38 minutes per week and starting with lower TG levels (**I**, **J**).

In order to validate the observations from the ExMet trial, we analysed heterogeneity and response to a 3-month supervised exercise program in the MetS sub-population of the OptimEx study (*n* = 34). Neither V̇O_2_peak, nor MetS-defining parameters changed significantly throughout exercise program (Figure 3A). Improvements in V̇O_2_peak during the ET program did not correlate with changes in MetS risk factors (Figure 3B) and were not associated with the ET modality (26). UMAP and GMM suggested three clusters of patients, which mainly differed in TG levels (*p* = 4.9e-07), SBP (*p* = 0.04) and FPG (*p* = 0.02), but not waist circumference (Figures 3C–G, Supplementary Figure S4). Recursive partitioning based on parameters of the training program and on MetS risk factors at inclusion suggested that—similar to the ExMET population—relative TG lowering was greater in participants with higher TG levels at baseline, albeit cut-off values were different in both studies (Supplementary Figure S5, Figure 3H). SBP and DBP reduction in the OptimEx MetS subpopulation was greater in individuals exercising for a longer duration per session and starting with lower plasma TG levels (Figures 3I,J). No difference between pre-specified exercise programmes (HIIT, MICT) on the change of cardiometabolic risk factors was observed (*data not shown*).

Both study populations investigated here fulfilled the MetS criteria as delineated by the IDF (1), but differed in a number of characteristics, most importantly the additional diagnosis of HFpEF in OptimEx. Furthermore, the MetS subpopulation of the OptimEx cohort had included more female participants, had lower DBP, but higher TG and lower V̇O_2_peak/kg at inclusion than the ExMET cohort (Table 1). Training programs reached comparable relative exercise intensities (% of individual heart rate reserve) and volumes per session, as well as adherence to the supervised sessions (participants with less than 70% adherence had been excluded in both studies), while the overall program length, the number of concluded sessions and cumulative duration of training per week (minutes), and therefore also the number of sessions and cumulative duration throughout the whole supervised program was higher in the ExMET study than in the OptimEx MetS population (Table 2). We did not analyse the unsupervised phase of the OptimEx study (3–12 months) (24, 28).

**Table 2:** Exercise parameters of ExMET and optimEx training programs.

	ExMET (*n* = 24)	OptimEx-MetS (*n* = 34)
Maximal intensity [%HRR]	67.2 (53.4–86.4)	74.8 (53.4–88.5)
Average intensity [%HRR]	56.2 (47.4–65.2)	59.9 (51.1–65.7)
Total number of sessions concluded per whole program [*n*]*	48.0 (48.0–71.3)	41.0 (31.5–53.0)
Total volume per whole program [%HRR*min]	95,474.5 (55,230.6–120,516.2)	87,663.5 (73,322.5–116,223.4)
Adherence per whole program [% of sessions concluded]	89.0 (81.0–94.3)	90.0 (77.0–97.0)
Average volume per week [%HRR * min]	6,594.1 (3,502.2–8,099.8)	6,678.2 (5,335.9–8,433.7)
Average number of sessions per week [*n*] *	3.0 (3.0–5.0)	2.9 (2.4–4.4)
Average duration per week [min]*	110.3 (57.3–121.5)	109.0 (92.0–172.8)
Average duration per session [min]*	30.0 (20.0–30.0)	38.2 (38.0–40.0)
Average duration of program [weeks]*	16.0 (16.0–16.0)	13.6 (12.9–15.0)

Values are median (IQR).

* *p* < 0.05 ExMET vs. OptimEx-MetS (Wilcoxon test).

### Changes in inflammatory parameters and cardio-metabolic markers during a 4-month exercise program

**Figure 4:**
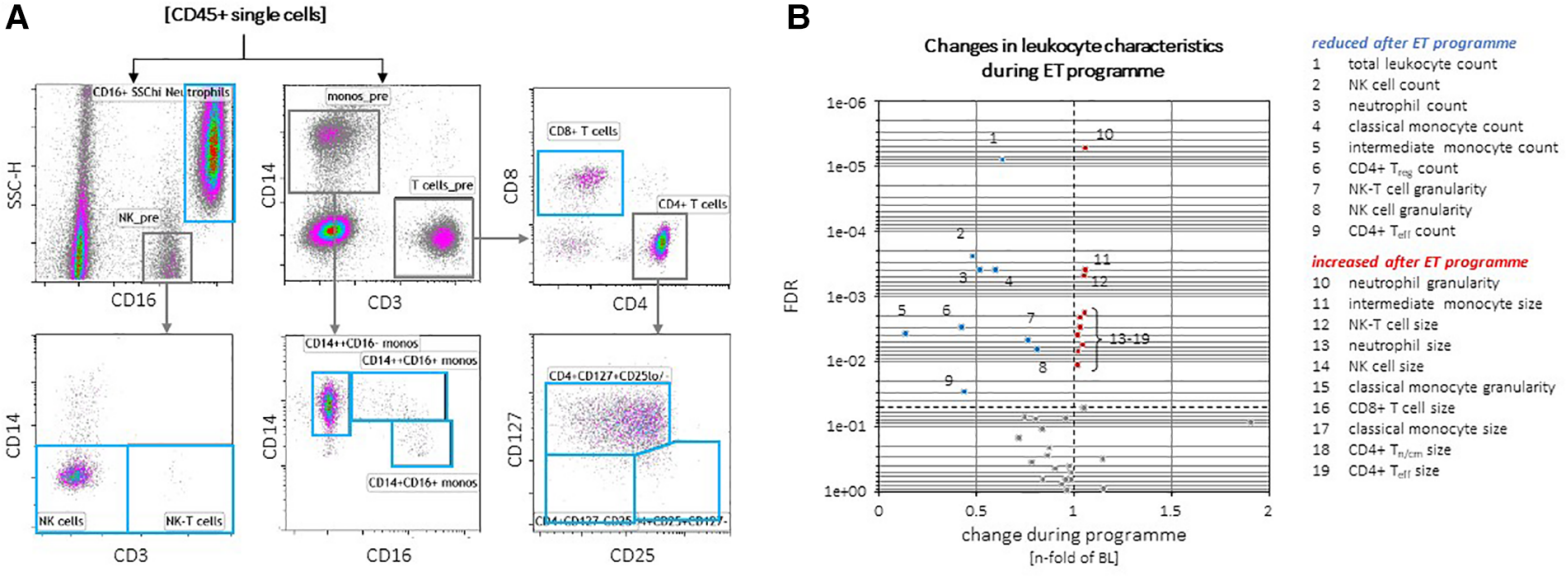
Exemplary gating of individual leukocyte populations (**A**). CD45^pos^ pan-leukocyte counts, as well as total, classical and intermediate monocytes, neutrophils and NK cell counts, granularity of NK and NK-T cells were reduced in the total ExMET cohort after 16 weeks (**B**). Cell sizes of classical monocytes, CD4^pos^ T_n/cm_ and T_eff_ cells, CD8^pos^ T cells, NK cells and neutrophils as well as granularity of classical monocytes were slightly increased at follow-up versus baseline (**B**).

**Figure 5:**
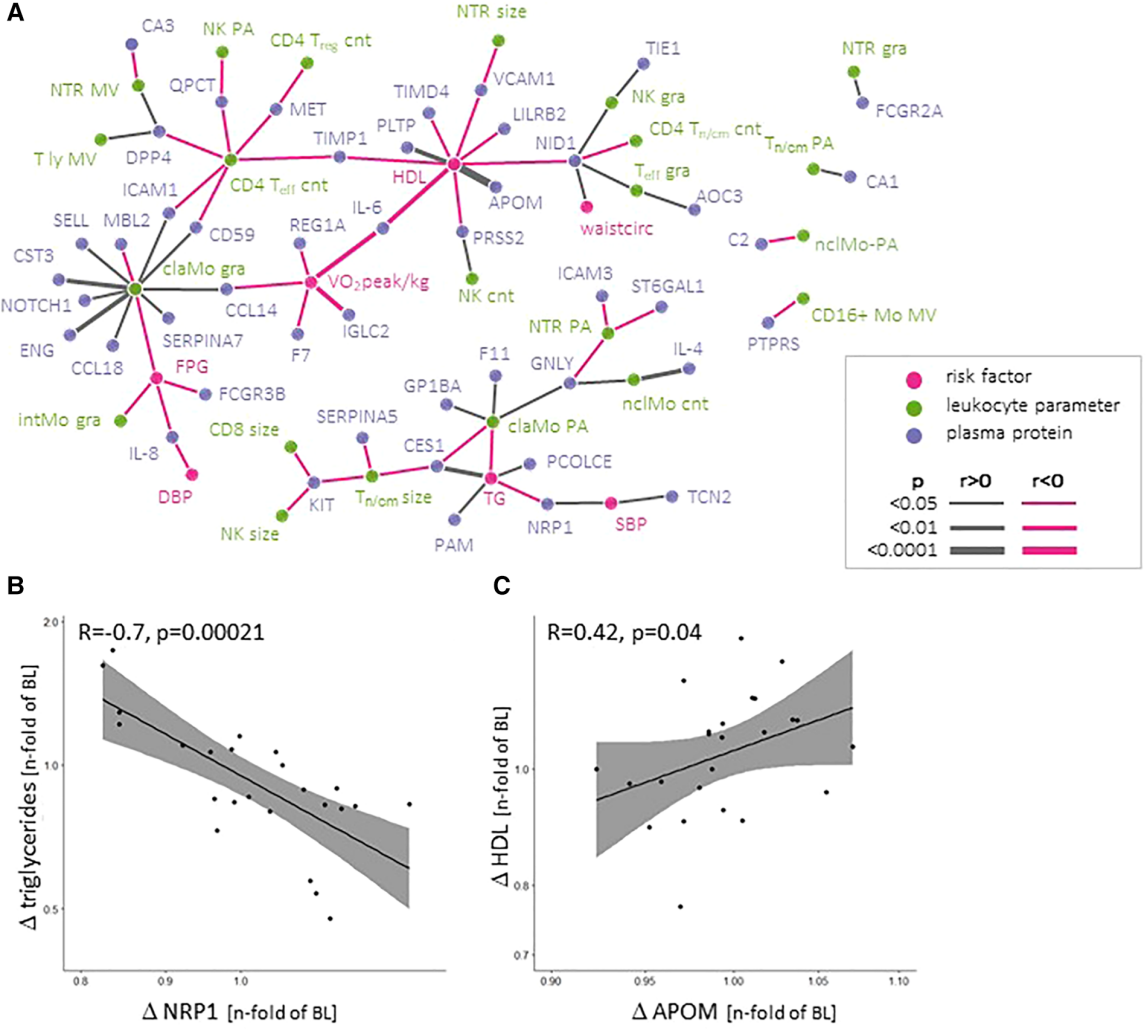
Network depicting correlations between absolute values of MetS-defining (clinical) parameters, incl. VO_2_peak, and leukocyte parameters and plasma proteins (**A**) Changes in soluble neuropilin-1 (NRP-1) and Apolipoprotein M (APOM) plasma levels correlate with changes in triglycerides and HDL-C during the program, respectively (**B**, **C**).

Across the entire ExMET population, an intra-individual reduction in absolute counts of total leukocytes, as well as sub-populations with effector functions, including CD25^neg^CD127^neg^CD4^pos^ effector (T_eff_) T-lymphocytes, CD14^hi^CD16^pos^ “intermediate” monocytes, natural killer (NK) cells and neutrophil granulocytes, was observed during the course of the training program (Figure 4). Throughout the ExMET cohort and covering both time points, absolute values of V̇O_2_peak correlated inversely with plasma levels of IL-6 and the immunoglobulin lambda constant 2 (IGLC2), suggesting a lower activation state of innate immunity in patients with higher V̇O_2_peak—independently of whether this was intrinsic or achieved during the exercise program (Figure 5A). Fasting plasma triglyceride levels correlated with circulating levels of the carboxyl esterase 1 (CES1) and the peptidylglycine alpha-amidating monooxygenase (PAM) (Figure 5A). HDL-C levels correlated with levels of apolipoprotein M and phospholipid transfer protein (PLTP) and correlated inversely with IL-6 and with soluble leukocyte immunoglobulin-like receptor B2 (LILRB2), among others (Figure 5A).

Absolute plasma levels of these proteins at both time points might be strongly influenced by intrinsic factors, including long-term lifestyle and epi-/genetic factors. We assumed that these co-variables remained stable within the same individual. To assess the effect of the exercise programme, we therefore correlated intra-individual changes between baseline and follow-up for plasma protein levels and MetS parameters. Only changes in NRP-1 levels were inversely associated with triglyceride changes (Figure 5B) and changes in plasma APOM levels (a component of HDL-C) were associated with plasma HDL-C levels (Figure 5C). No differences in the change of individual plasma proteins or cytokines between pre-defined exercise groups or exercise parameters were observed (*not shown*).

## Discussion

Our exploratory analysis of the heterogeneity in response to a 3-to-4-month exercise program among patients with MetS revealed three main findings:

(1) Improvement of cardiorespiratory fitness during the exercise program did not consistently correlate with improvements in individual MetS risk factors.
(2) The effect of exercise on individual MetS-relevant risk factors differed with the patient-specific risk factor profile in combination with the exercise parameters. Within the conditions applied in our programs and the populations we studied, exercise consistently supported a reduction in triglycerides when basal triglyceride levels were higher and a reduction in SBP and DBP when basal triglyceride levels were lower and exercise durations per week or per session were higher.
(3) A 4-month endurance exercise training lowered leukocyte blood counts, especially effector subtypes, without detectable differences between participants with different risk factor profiles at baseline, or exercise parameters.

### Improvement in V̇O_2_peak as a surrogate for improvement in cardiovascular risk factors?

V̇O_2_peak and its change over time is acknowledged as a prognostic marker of survival (30, 31). Physical fitness can be improved by ET, as can MetS-associated risk factors, including hypertension (32). By extension, exercise-induced increase of V̇O_2_peak is often used as a surrogate of improvement in overall morbidity improvement by ET, including cardiovascular or metabolic risk factors and has been used as a primary endpoint in interventional studies. Our data underline that this intuitive association must be reviewed with caution. The association of change in V̇O_2_peak and change in cardiovascular risk profile in observational studies may be affected by long-term (voluntary) individual lifestyle/habitually high physical activity and may reflect a behavioural selection process (31, 33). The same association may not necessarily be transferable to a prescribed exercise training program of a few weeks or months in a population with decades-long low habitual physical activity and with potential differences in genetic links between behaviour and metabolism and in neural reward circuits (34, 35).

In our analysis of two RCTs with populations of MetS patients, we did not observe associations between the extent of increase in V̇O_2_peak with improvement in cardio-metabolic risk factors, except for a weak association between DBP reduction and increase in V̇O_2_peak within the ExMET, but not the OptimEx-MetS population. Of note, DBP at baseline was higher in ExMET than in OptimEx-MetS. This observation is in line with a meta-analysis of 72 trials, which observed greater reduction of resting BP in hypertensive study groups than in controls (32). Recursive partitioning analyses did not indicate relevance of a change in V̇O_2_peak for the extent in DBP change during the ExMET or the OptimEx training programs.

Taken together, the extent of improvement in V̇O_2_peak might correlate with the extent in the lowering of individual cardio-metabolic risk factors in some patient populations, but this is not a universal association. Notably, we have assessed two studies with rather different patient characteristics. In our view, the similar findings despite the differences between the study populations further strengthen the notion to carefully re-assess/re-consider the undisputed suitability of V̇O_2_peak change as a surrogate of change in individual cardiometabolic risk factors.

### Reduced inflammatory load by exercise training

The benefit of endurance training for the global reduction in inflammatory status is well established in the literature (18). However, it remained unclear whether these effects universally apply for MetS patients with different individual risk factor profiles. Moreover, previous studies often view inflammation as a one-dimensional parameter, assessing single downstream parameters (e.g., plasma CRP) or overall leukocyte counts, or they focus only on selected leukocyte subsets or pathways (e.g., T_reg_ lymphocytes). By mapping changes in the array of leukocyte subsets of the same MetS individuals before and after the training period, we aimed to understand potential shifts in the combined spectrum of circulating leukocytes.

Our study expands the current knowledge by the following two aspects: First, we report that especially effector subtypes such as CD127^neg^CD25^neg^CD4^pos^ T effector cells, NK cells and neutrophils decreased during the exercise program. Second, we observed no association between the extent of improvement in V̇O_2_peak and extent of leukocyte reduction, nor between leukocyte changes and exercise protocol or individual exercise parameters. The latter observation indicates that cardiorespiratory fitness and inflammatory status should be both considered separately when evaluating the response to ET in patients with MetS.

Circulating leukocyte counts are known to be higher in patients with increased cardiovascular risk and with MetS (36, 37) and lower in regularly exercising individuals (38). Albeit we do not have a “healthy” control group, our observations may be interpreted in light of these reports: circulating numbers of leukocytes were likely elevated at baseline and we have observed a decrease when our patients engaged in regular training. Mechanistic studies have reported a reduction of leptin-mediated production of haematopoietic stem cells and their subsequent release into the circulation (39). By extension, in our human population, we have observed especially effector cell types to be reduced over the training period. One potential explanation might be a lower availability of metabolic ligands due to the regular training. Modified metabolites, including advanced glycated end-products and oxidized phospholipids and low-density lipoproteins (LDL)—elevated in individuals with high plasma levels of glucose and LDL-C—serve as ligands to pattern recognition receptors and aggravate inflammatory processes (40–42). We would therefore speculate that an exercise-mediated lower concentration of these ligands might result in lower innate effector cell activation (and granularity) and potentially allow for the cell to spend a longer time in the circulation without activation (e.g., phenotype shift).

Several aspects might modulate the effects of an exercise intervention on the interactions between obesity and inflammation. Exercise intensity and the duration of the training programme crucially mediate changes in fat mass and leukocyte counts, with higher exercise intensities, resistance training and longer training programmes achieving greater effects (43–45). In addition, concurrent lifestyle habits, including leisure time physical activity and sedentary behaviour and diet, impact on the energy balance and metabolic signalling. Notably, the duration of time spent sitting is an independent metabolic risk factor, with long sitting times affecting post-prandial glucose handling and lipogenesis (46).

### Effects on MetS-defining risk factors

In contrast to the effects on leukocyte parameters, ET did not lead to a global reduction in waist circumference, plasma levels of triglycerides, SBP/DBP, nor to an overall increase of HDL-C in the two RCTs we have analysed. Instead, exercise predominantly affected triglycerides, SBP, DBP and FPG in participants where those parameters were elevated at baseline, and in combination with a minimum exercise duration. These changes were not explained by altered medication. Of note, our programs did not include a dietary component, which might have supported reduction in central adiposity/waist circumference (17). No correlation with the baseline values was observed for changes in waist circumference or HDL-C, nor for V̇O_2_peak.

Our observations underline the concept of MetS as an accumulation of heterogeneous cardiometabolic risk factors where the specific risk factor constellation will differ—by definition—between individual patients and might potentially affect the efficacy of the training program and underlying molecular and cellular processes. Our clustering approach mirrors this concept and expands the current knowledge on the interaction between individual risk factors evident at baseline and the exercise-mediated improvement of other risk factors through the ET program. Within our study population, individual clusters emerged with elevated triglyceride levels as the respective predominant risk factor, and DBP, SBP and FPG contributing to a smaller extent. Of note, baseline triglyceride status also impacted on the improvement in SBP and DBP.

### Effects on plasma proteins with cardio-metabolic relevance

None of the tested 86 plasma proteins with cardiometabolic relevance changed across the entire ExMET population during the ET program. Our study newly underscores the potential role of NRP-1 in monitoring and/or modulating triglyceride levels in patients with MetS. The soluble form of the growth factor co-receptor NRP-1 was inversely correlated with fasting plasma triglycerides and also the changes in NRP-1 and triglyceride plasma levels during the ET program showed an inverse correlation. NRP-1 serves as a co-receptor to several growth factor (GF) receptors, including vascular endothelial, the hepatocyte and the platelet-derived GF (VEGF, HGF, PDGF, respectively) receptors, all of which are potentially involved in metabolic diseases (47). A strong role of NRP-1 has been described in vascular biology, including angiogenesis and the development of atherosclerosis (48, 49). Circulating NRP-1 fragments may derive from alternative splicing or proteolytic cleavage and may affect vascular function (47, 50–52). NRP-1 might also potentially regulate the interaction of metabolic and inflammatory processes. The VEGF receptor 1 and NRP-1 cooperatively regulate organ-specific lipid storage (53). Additional data indicate NRP-1 intracellular trafficking, its location at the mitochondrial membrane and involvement in mitochondrial function (54, 55). NRP-1 expressed by adipose tissue macrophages shifts the inflammatory phenotype in adipose tissue to a more protective spectrum (56). Hence, differential NRP-1 processing—i.e., the generation of soluble and intracellular fragments with distinct signalling characteristics via alternative splicing and proteolytic degradation, reflected by soluble NRP-1 plasma levels—might link to the individual MetS phenotype represented by individual cardiac, vascular and metabolic risk components. A better understanding of the underlying molecular interactions might help to develop targeted therapies to modulate lipid processing and dampen adipose tissue inflammation, as well as biomarker-based risk monitoring to guide therapy decisions. This is in line with recent network analyses in familial dyslipidaemia and in HFpEF which both returned NRP-1 as a putative target (57, 58). The only other protein—Apolipoprotein M—whose absolute levels and changes throughout the ET program correlated with HDL-C levels and changes, respectively. This might simply reflect the presence of Apo M as a component of the HDL particle.

### Limitations

The interpretation of our data requires consideration of a number of aspects. This analysis comprises a low number of patients, which is a frequent problem of exercise trials that necessitate greater efforts in coordination and personnel cost, greater continuous motivation of participants and greater commuting and coordination efforts for outpatients in supervised programs. Our study comprised two different populations of patients with MetS. While the ExMET study aimed to investigate MetS patients, this was not the pre-specified aim of the OptimEx trial. Vice versa, presence of HFpEF was not pre-specified in ExMET. Hence, patient characteristics differed between both sub-populations. No “sedentary” control group is included in the design of the ExMET study since current guidelines recommend moderate intensity endurance training in these patients. While MICT and high volume HIIT groups were comparable between OptimEx-MetS and ExMET, the low volume HIIT group was unique to ExMET. Between these pre-specified exercise groups, no differences in the change of individual parameters were observed, probably due to low numbers of patients per group.

While we assume that the exercise training programme was causal for the changes observed, it is possible that participants modified their physical activity patterns outside the training sessions. Two independent factors might play role here: (a) study participants reducing their habitual exercise because of exercising during the study and (b) study participants extending periods of uninterrupted sitting, e.g., “resting”/viewing TV in their leisure time (59–61). These independent parameters might be acquired using wearable activity trackers in future studies.

We cannot exclude regression to the mean to play a role in the association between TG levels at baseline and their reduction throughout the program. An association of reduction with baseline levels was only observed for fasting TGs, not for the other parameters assessed. In the small (*n* = 17) subgroup of MetS patients within the control group of the OptimEx study not participating in supervised training, no such effect was observed (patients with TG <171.8 mg/dl at baseline: mean reduction of 4%, patients with TG >171.8 mg/dl at baseline: mean reduction of 5% vs. baseline, *p* = n. s.).

In conclusion, our data support the anti-inflammatory role of exercise in patients with MetS. Individual changes in the heterogeneous patient-specific risk factor profiles were independent from changes in inflammation and changes in V̇O_2_peak and should therefore be considered separately when assessing the influence of exercise training. In addition, soluble NRP-1 may be a new potential biomarker and tool to monitor therapy progress, especially in the subgroup of patients with elevated fasting triglycerides. Future studies are warranted to explore underlying mechanisms and identify druggable targets in this subgroup of patients with triglyceride-dominated cardiovascular risk.

## Data Availability

The raw data supporting the conclusions of this article will be made available by the authors, without undue reservation.
