# Supplementary material for: Cardiometabolic and immune response to exercise training in patients with metabolic syndrome: retrospective analysis of two randomized clinical trials
Source: Front Cardiovasc Med. 2024 Apr 3;11:1329633. doi: 10.3389/fcvm.2024.1329633 (PMC11025358; doi:10.3389/fcvm.2024.1329633)
Supplement: Supplementary file 1 [file Datasheet1.pdf]

Supplemental Material

Cardiometabolic and immune response to exercise training in patients with metabolic syndrome: Retrospective analysis of two randomized clinical trials

Katharina Lechner[1,2,3*], Sylvia Kia[4,5*], Pia von Korn[1,2*], Sophia M Dinges[1,2], Stephan Müller[1,2], Arnt-Erik Tjønna[6], Ulrik Wisløff[6,7], Emeline M Van Craenenbroeck[8,9], Burkert Pieske[10], Volker Adams[11], Axel Pressler[1,2,12], Ulf Landmesser[4,5,13], Martin Halle[1,2], Nicolle Kränkel[4,5,13,§]

1 Department of Prevention and Sports Medicine, School of Medicine, Technical University of Munich, Munich, Germany.
2 DZHK (German Centre for Cardiovascular Research), Partner site Munich Heart Alliance, Munich, Germany.
3 Klinik für Herz- und Kreislauferkrankungen, Deutsches Herzzentrum München, Technische Universität München, Munich, Germany.
4 Deutsches Herzzentrum der Charité, Klinik für Kardiologie, Angiologie und Intensivmedizin, Campus Benjamin-Franklin (CBF), 12203 Berlin, Germany
5 DZHK (German Centre for Cardiovascular Research), Partner site Berlin, Germany
6 Cardiac Exercise Research Group (CERG), Department of Circulation and Medical Imaging, Norwegian University of Science and Technology, Trondheim, Norway.
7 Centre for Research on Exercise, Physical Activity and Health, School of Human Movement and Nutrition Sciences, University of Queensland, St. Lucia, Brisbane, Queensland, Australia.
8 Research Group Cardiovascular Diseases, University of Antwerp, B-2610 Antwerp, Belgium
9 Department of Cardiology, Antwerp University Hospital (UZA), Drie Eikenstraat 655, B-2650 Edegem, Belgium.
10 Berlin, Germany
11 Heart Center Dresden-University Hospital, Department of Cardiology and Internal Medicine, TU Dresden, Dresden, Germany.
12 Private Center for Sports and Exercise Cardiology, Törringstr. 6, 81675 Munich, Germany
13 Friede Springer - Centre of Cardiovascular Prevention @ Charité, Charité University Medicine Berlin, Berlin, Germany

* Equal contribution

§ correspondence to: Nicolle Kränkel, PD Dr. rer. nat., Dipl.-Ing. (FH)
Deutsches Herzzentrum der Charité
Hindenburgdamm 30A
12203 Berlin
Germany
ph.: +49-30-450 522256
email: nicolle.kraenkel@dhzc-charite.de

Supplemental Research design and Methods

Patient inclusion, clinical measurements, and exercise program

ExMET cohort: Twenty-nine patients fulfilling IDF criteria for MetS were randomized (1:1:1) to a moderate intensity continuous training (MICT), low-, or high-volume high intensity interval training (HIIT) with four months duration (**suppl. Fig. 1**). MICT was defined as two supervised sessions of 30 minutes continuous aerobic training at 35–50% heart rate reserve and three home-based exercise sessions, according to current guideline-based standard of care. Low-, and high-volume HIIT were defined as a 10-minutes warm-up phase at 35–50% HRR, followed by a four-minute interval at 80%–90% HRR for the low-volume HIIT-group and four four-minute intervals for the high-volume HIIT-group in a supervised setting. The detailed protocol has been published and the study has been completed.[1] Baseline and follow-up visits included a medical examination, anthropometric measurements, laboratory testing and standardized cardiopulmonary exercise testing (CPET). Venous blood samples were drawn from an antecubital vein after 12 hours of fasting and after 24 hours of abstinence from alcohol and vigorous exercise. The study investigators did not change medication (as prescribed by the treating physician) at baseline and patients were encouraged to take medications as prescribed throughout the study period.

OptimEx-MetS sub-cohort: Of the n=180 patients included into the completed OptimEx trial[2], n=35 patients meeting the IDF criteria for MetS [3] and with data of baseline and 3-month visit complete were included into this analysis. Patients were included in the OptimEx trial if heart failure with preserved ejection fraction (HFpEF), based on elevated natriuretic peptides, left ventricular ejection fraction of ≥50% and increased estimated left ventricular filling pressure[4] were evident and signed informed consent obtained. Examination at baseline and three months-visit included, amongst others, medical history, anthropometric measurements, blood sampling and CPET.[2]

Leukocyte profiling

One hundred µL of freshly withdrawn, EDTA-anticoagulated blood was stained with directly fluorochrome-labelled antibodies (CD45-Brilliant Violett 711; CD3-PE/Cy7; CD14-Brilliant Violett 421; CD8-Brillian Violett 510, CD16-Alexa Fluor 647; CD4-AlexaFluor 488; CD25-Alexa Fluor 700, CD127-Brilliant Violett 605; CD41-APC/Cy7) and fixed in 1% PFA after a 20-minute incubation in the dark. Samples were measured within three days and diluted 5-fold in PBS prior to acquisition on an Attune NxT acoustic focusing cytometer. Analysis was performed using Kaluza Flow Cytometry Analysis software (Beckman Coulter). The person acquiring the data and performing the gating was blinded to group assignment. Data were shared with the cooperation partner and time-stamped before unblinding.

Microvesicle measurements

Freshly withdrawn acid citrate dextrose (ACD)-anticoagulated blood was centrifuged for 15 min at 1500 x rcf. The upper ca. four fifth of clear plasma were transferred into a fresh tube and again centrifuged at 2000 x rcf for 20 min to further deplete thrombocytes. The upper four fifth of the supernatant were frozen at -80°C until analysis. For analysis, plasma samples were thawed once and centrifuged again at 2000 x rcf for 20 min. A master mix of directly fluorochrome-labelled antibodies (CD3-PE/Cy7, CD4-Alexa Fluor 488, CD8-BV510, CD14-BV421, CD16-Alexa 647, CD41-APC/Cy7, CD45-BV711, CD66b-Alexa Fluor 700, CD144-PE, all BioLegend) and Annexin V-PerCP/Cy5.5 was prepared in

Annexin V Binding Buffer (BioLegend) supplemented with $CaCl_2$ to a final concentration of 20mM and centrifuged at 16.000 x rcf at 4°C for 20 min to remove antibody aggregates. 200µL of cleared plasma were combined with 50µL of cleared antibody master mix and incubated in the dark for 20 min. 400µL of Annexin V Binding Buffer were added and samples acquired immediately on an Attune NxT acoustic focusing cytometer. The person acquiring the data and performing the gating was blinded to group assignment. Data were shared with the cooperation partner and time-stamped before unblinding.

Plasma measurements

Concentrations of cytokines (interleukin (IL)-1β, IL-8, IL-10, IL-13, basic fibroblast growth factor (FGF), interferon (IFN)-γ, IFN-γ induced protein (IP)-10, monocyte chemoattractant protein (MCP)1, tumor necrosis factor (TNF)-α) were assessed in cleared EDTA plasma samples of the ExMET cohort using the respective cytokine bead array (CBA) flex kits (BD) according to the protocol recommended by the manufacturer and read in Attune NxT acoustic focusing cytometer. The person acquiring the data and performing the gating was blinded to group assignment.
A panel of 86 plasma proteins relevant to cardiometabolic disease was assessed in cleared EDTA plasma samples of the ExMET cohort using proximity extension assay by Olink© Proteomics (Uppsala, Sweden). The service provider was blinded to group assignment and data provided by the service provider are time-stamped.
Apolipoprotein C3 plasma levels were assessed in cleared EDTA plasma samples from the OptimEx-MetS-cohort using the Apolipoprotein C3 Human ELISA Kit (Invitrogen) as recommended by the manufacturer. The person performing the ELISA was blinded to group assignment.

Macrophage polarization

Human monocytic THP-1 cells were grown in RPMI medium, exposed to 10% ExMET patient´s ACD plasma in RPMI for three days, treated with PMA for 2 days and subsequently stimulated for 24h with lipopolysaccharides (LPS, 10ng/mL) & IFN-γ (100ng/mL), IL-4 (10ng/mL) or IL-10 (10ng/mL) for polarization into M1, M2a and M2c macrophages, respectively, as described by Genin et al., all in the continued presence of patients´ plasma.[5] Control samples with heat-inactivated fetal calf serum instead of patients' plasma and without cytokine stimulation were maintained in parallel. Median cell surface expression of CD86, CD163 and CD206 were determined by flow cytometry. The person acquiring the data and performing the gating was blinded to group assignment.

Statistical analysis

Parameters or participants with more than 10% missings of the respective parameters were omitted from the analysis. Excel 2016 (Microsoft) was used to compile data and R version 3.6.0 with R Studio version 1.1.463 was used for analysis and drawing of graphs. Non-parametric tests were used at all times due to the low n. The Benjamini-Hochberg method was used to adjust for multiple testing where false discovery rate (FDR) is given. A p-value of less than 0.05 was considered statistically significant. "Delta" values (Δ) denote n-fold changes at the 3-months or 4-months follow-up time point, normalized to the baseline value of the same individual.
Uniform manifold approximation and projection (UMAP, R package *umap* version 0.2.10.2) was performed using MetS criteria of participants at baseline following imputation of missings by Multivariate Imputation by Chained Equations (R package *mice* version 3.15.0). Gaussian mixture model (GMM, R package *umap* version 0.2.10.2) was used to identify clusters. Recursive partitioning

(R package *rpart* version 4.1.19) was applied to explore the impact of MetS parameters at baseline and training conditions on the change of individual MetS parameters.

Tables

supplemental Table I: ExMET Patients' medication at baseline.

medication	% of patients medicated
ARB (%)	41.7
Statins (%)	45.8
beta blockers (%)	29.2
ASS (%)	29.2
oral antidiabetics (%)	8.3
Insulin (%)	8.3

supplemental Table II: OptimEx MetS patients' medication at baseline.

medication	% of patients medicated
ACEi/ARB (%)	91.2
Statins (%)	52.9
other lipid lowering drugs (%)	2.9
beta blockers (%)	73.5
ASS (%)	35.3
NOACs (%)	20.6
vitamin K antiagonists (%)	11.8
other anti-platelet drugs (%)	8.8
oral antidiabetics (%)	32.4
Insulin (%)	17.6

Supplemental Figures

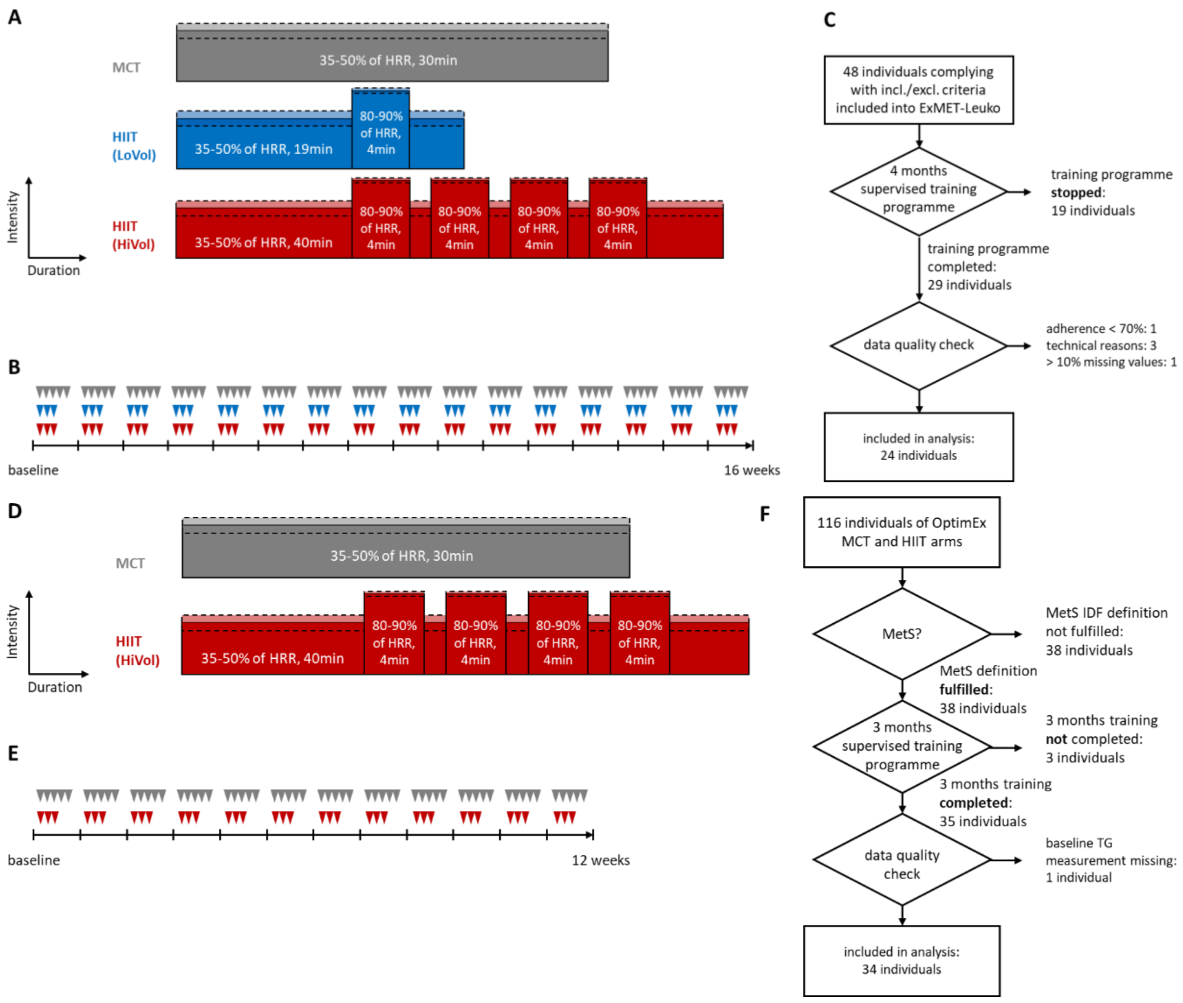

supplemental Figure 1: Depiction of exercise programs regarding intensity and duration of a single session (ExMET study **A**, OptimEx-CLIN trial **D**) and frequency per week and program duration (ExMET study **B**, OptimEx-CLIN trial **E**). Flow chart detailing patient inclusion and drop-out (ExMET study **C**, OptimEx-CLIN trial **F**).

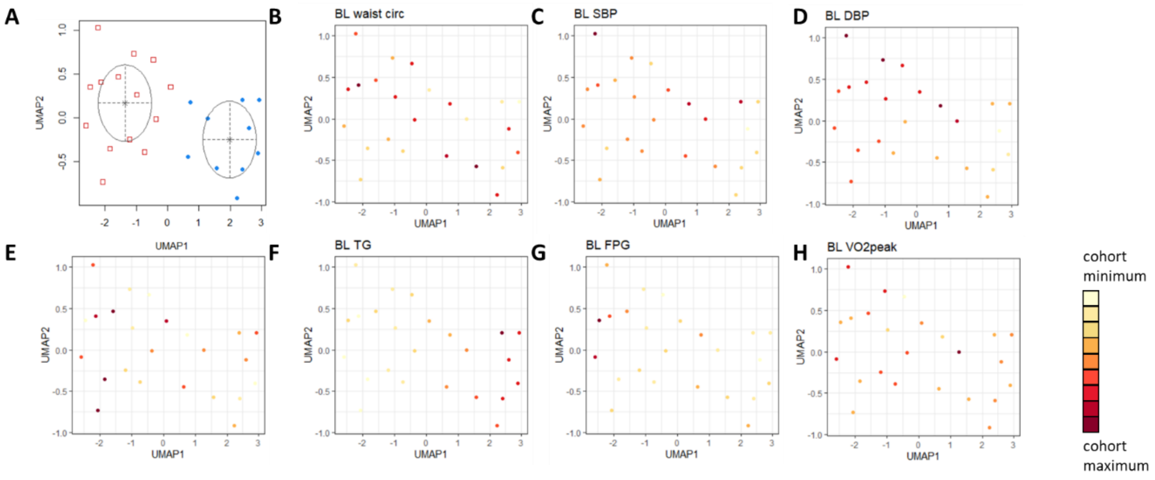

supplemental Figure 2: MetS risk factors at inclusion were used to cluster participants of the ExMET study for similarity by UMAP. GMM identified 2 clusters (**A**) which mainly differed by TG levels (p=4.64e-05)(**F**) and DBP (p=0.01804)(**D**).

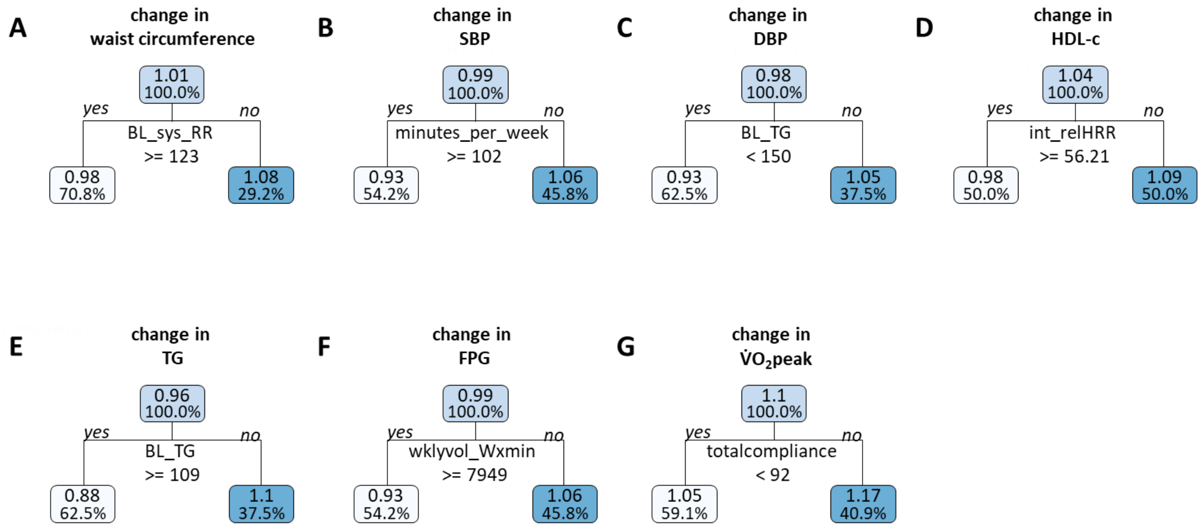

supplemental Figure 3: Recursive partitioning was applied to understand which MetS risk factors at inclusion and which parameters of the ExMET training programs determine benefit in the individual MetS parameters during the exercise program.

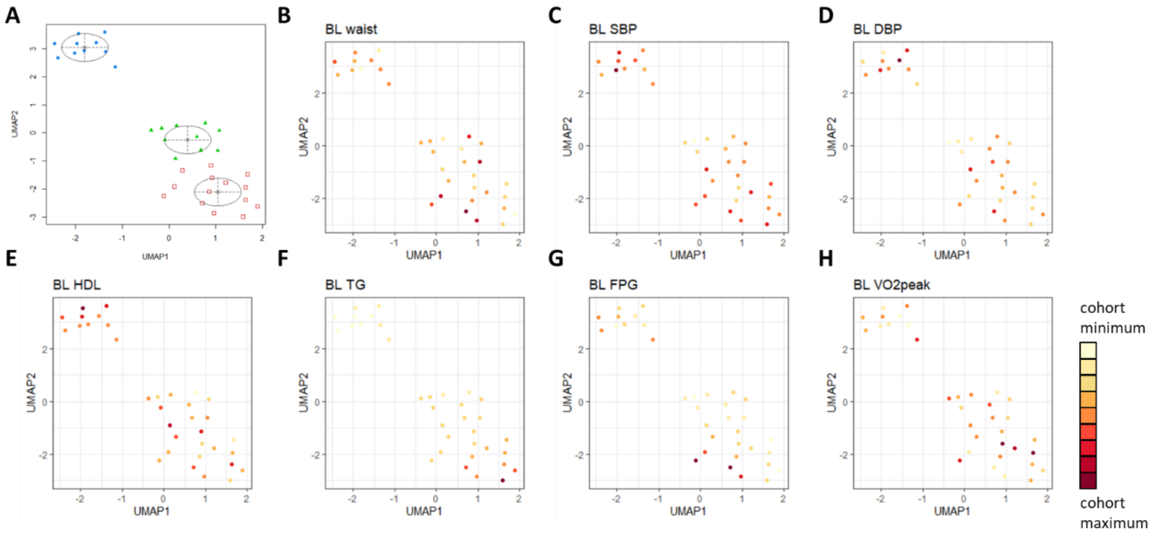

supplemental Figure 4: MetS risk factors at inclusion were used to cluster participants with MetS of the OptimEx-CLIN trial for similarity by UMAP. GMM identified 3 clusters (**A**) which mainly differed by SBP (p=0.04192)(**C**). fasting TG levels (p=4.895e-07)(**F**) and FPG (p=0.02428)(**G**).

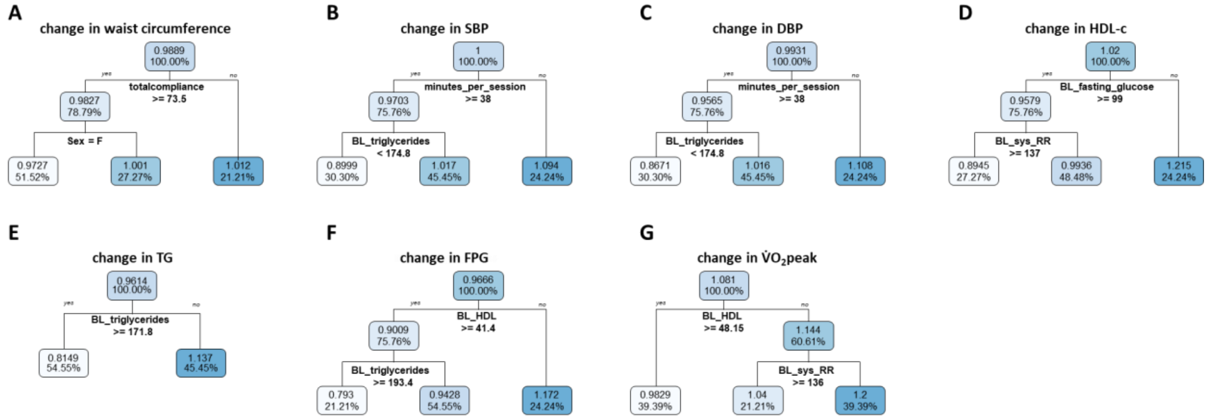

supplemental Figure 5: Recursive partitioning was applied to understand which MetS risk factors at inclusion and which parameters of the OptimEx-CLIN training programs determine benefit in the individual MetS parameters during the exercise program.

References

1. von Korn P. Keating S. Mueller S. *et al.* The Effect of Exercise Intensity and Volume on Metabolic Phenotype in Patients with Metabolic Syndrome: A Randomized Controlled Trial. *Metabolic Syndrome and Related Disorders* 2020;**19**:107-114. doi: 10.1089/met.2020.0105

2. Mueller S. Winzer EB. Duvinage A*. et al.* Effect of High-Intensity Interval Training. Moderate Continuous Training. or Guideline-Based Physical Activity Advice on Peak Oxygen Consumption in Patients With Heart Failure With Preserved Ejection Fraction: A Randomized Clinical Trial. *Jama* 2021;**325**:542-551. doi: 10.1001/jama.2020.26812

3. Alberti KG. Zimmet P. Shaw J. Metabolic syndrome--a new world-wide definition. A Consensus Statement from the International Diabetes Federation. *Diabet Med* 2006;**23**:469-480. doi: 10.1111/j.1464-5491.2006.01858.x

4. Paulus WJ. Tschope C. Sanderson JE*. et al.* How to diagnose diastolic heart failure: a consensus statement on the diagnosis of heart failure with normal left ventricular ejection fraction by the Heart Failure and Echocardiography Associations of the European Society of Cardiology. *Eur Heart J* 2007;**28**:2539-2550. doi: 10.1093/eurheartj/ehm037

5. Genin M. Clement F. Fattaccioli A. Raes M. Michiels C. M1 and M2 macrophages derived from THP-1 cells differentially modulate the response of cancer cells to etoposide. *BMC Cancer* 2015;**15**:577. doi: 10.1186/s12885-015-1546-9
